# Deviation from Normal Values of Leukocyte and Erythroblast Parameters in Complete Blood Count is a Messenger for Platelet Abnormalities

**DOI:** 10.4274/Tjh.2012.0170

**Published:** 2014-03-05

**Authors:** Cengiz Beyan, Kürşat Kaptan

**Affiliations:** 1 Gülhane Military Medical Academy, Department of Hematology, Ankara, Turkey

**Keywords:** Blood cell count, Blood platelets, Blood platelet disorders, Peripheral blood smear

Automated blood cell counters have undergone a formidable technological evolution owing to the introduction of new physical principles for cellular analysis and the progressive evolution of software [1,2]. The results have been an improvement in analytical efficiency and an increase in information provided with new parameters. 

A 61-year-old male patient had the diagnosis of diffuse large B-cell lymphoma 6 years ago, and after chemotherapy, he was still in remission. He was hospitalized for high fever, fatigue, acute renal failure, and bibasilar crepitant rales. Complete blood count measured with a Beckman Coulter LH 780 hematology analyzer revealed an uncorrected leukocyte count (UWBC) of 63.5x10^9^/L, leukocyte count (WBC) of 22.1x10^9^/L, erythroblast count (NRBC) of 21.4x10^9^/L, and platelet count of 197x10^9^/L (Figure 1). Upon peripheral blood smear examination, we detected 5% neutrophils, 22% band forms, 61% metamyelocytes, 5% myelocytes, 1% promyelocytes, 2% myeloblasts, 2% lymphocytes, and 2% eosinophils. We also detected rare erythroblasts and large platelets with profuse platelet clumps (Figures 2 ). Routine biochemical analysis revealed high fasting glucose, blood urea nitrogen, creatinine, serum glutamic oxaloacetic transaminase, alkaline phosphatase, direct and indirect bilirubin, albumin, and lactate dehydrogenase. The erythrocyte sedimentation rate was 100 mm/h, and serum ferritin was 2944 ng/mL. High-resolution computed tomography of the thorax revealed bilateral diffuse infiltrations, nodular opacities, right pleural effusion, and mediastinal lymphadenopathies. Clarithromycin and imipenem/cilastatin were administered for a probable diagnosis of pneumonia. Bone marrow examination revealed myeloid hyperplasia but nothing else significant. No endobronchial mass was detected in bronchoscopy, but mucopurulent secretion was present in the right upper and lower lobes. Biopsy reports showed non-neoplastic bronchial mucosa epithelium. Sputum, blood, and urine cultures; sputum mycobacterial examination; and serum galactomannan antigen were all negative. After the general condition, fever, acute renal failure, signs, and symptoms were relieved, the patient was discharged. Informed consent was obtained.

When we subtracted the WBC and NRBC from the UWBC (=20.0x10^9^/L), a significanT-cell group was composed of big platelets. It is probable that this ratio was higher than calculated. Rare erythroblasts in the peripheral blood smear with high NRBC values support the idea of large platelets as cellular origin. In fact, the peripheral blood smear revealed large, profuse platelet clumps, contradictory to the platelet count. We conclude that complete blood counts should be examined carefully; despite the essential role of automation in the modern hematology laboratory, microscopic control of pathologic samples (i. e. peripheral blood smear) remains indispensable, so much so that in certain cases, it alone is diagnostic.

## Figures and Tables

**Figure 1:**
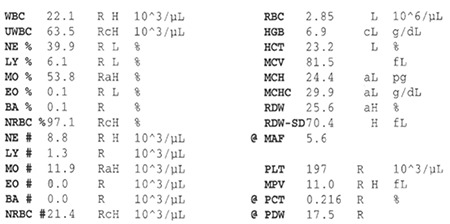
Complete blood count.

**Figure 2:**
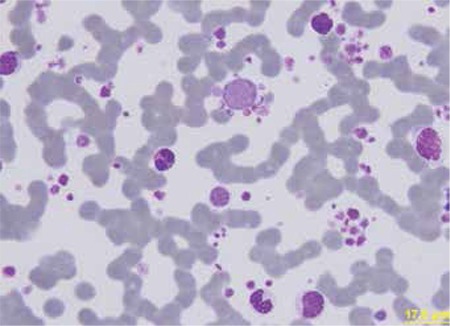
Peripheral smear. Large platelets with profuse platelet clumps are noteworthy.
